# Introduction to a special series: What Makes Man Human

**DOI:** 10.1186/1747-5333-1-12

**Published:** 2006-11-28

**Authors:** Neil R Smalheiser

**Affiliations:** 1 Department of Psychiatry and Psychiatric Institute MC912, University of Illinois-Chicago, 1601 W. Taylor Street, Chicago, IL 60612 USA

## Abstract

One of the most pressing and timely scientific questions concerns the evolution of man. In 1970, Karl Pribram delivered the James Arthur Lecture at the American Museum of Natural History in New York City. His lecture, "What Makes Man Human," was one of the most eloquent and brilliant syntheses of this problem ever made. The *Journal *is proud to publish this Lecture for the first time in an open access format that will make its insights available widely to a new generation of students and investigators. Accompanying the lecture is a new commentary written by Prof. Pribram, and four additional commentaries from prominent investigators who were invited to consider the question from their own perspectives. Together, these articles provide a scholarly, yet accessible, snapshot of different approaches to the study of human evolution in 2006.

As the Editorial that launched this journal states, "The *Journal of Biomedical Discovery and Collaboration *was created to provide, for the first time, a unified forum to consider all factors that affect scientific practice and scientific discovery – with an emphasis on the changing face of contemporary biomedical science." To date, the *Journal *has published a wide range of articles including historical case studies of scientific innovation, bibliometric analyses of scientific collaboration, and descriptions of informatics tools. Yet one of the most important factors driving scientific discovery is strategic – choosing new paradigms and model systems that can assist in solving an entire class of scientific problems. The Human Genome Project is a recent example of how one can strategically reformulate an existing field (genetics) into something new (genomics). The *Journal *intends to serve as a forum for articles that propose and discuss new strategic approaches to scientific investigation.

In this regard, one of the most pressing and timely scientific questions concerns the evolution of man. In 1970, Karl Pribram delivered the James Arthur Lecture at the American Museum of Natural History in New York City. His lecture, "What Makes Man Human," was one of the most eloquent and brilliant syntheses of this problem ever made, and it reads as well today as it did then, but was never published except as a pamphlet available as a hard-copy from the Museum. The *Journal *is proud to publish this Lecture for the first time in an open access format that will make its insights available widely to a new generation of students and investigators. Accompanying the lecture is a new commentary written by Prof. Pribram, who reassesses and updates his article in light of his more recent research and current thinking.

Moreover, several prominent investigators were invited to consider the question "What Makes Man Human" from their own perspectives, as well as from the vantage point of 2006. Prof. Ian Tattersall's commentary discusses the human fossil record. Andre Goffinet points out the importance of studying comparative neuroembryology across different vertebrate classes, whereas Todd Preuss emphasizes the importance of studying chimpanzees and other great apes. Nitzan Mekel-Brobrov and Bruce Lahn review the current state of comparative genomics for its prospects in understanding human evolution. All contributions were rigorously peer-reviewed by anonymous reviewers who were not commentators. Together, these articles provide a scholarly, yet accessible, snapshot of different approaches to the study of human evolution. May we never cease to ask ourselves the question "What Makes Man Human"!

